# Frequency of positive states of mind as a moderator of the effects of stress on psychological functioning and perceived health

**DOI:** 10.1186/2050-7283-1-13

**Published:** 2013-08-15

**Authors:** Richard Bränström

**Affiliations:** Department of Clinical Neuroscience and Department of Public Health Sciences, Karolinska Institute, Stockholm, 171 77 Sweden

**Keywords:** Perceived stress, Depression, Anxiety, Positive states of mind, Perceived health

## Abstract

**Background:**

Emerging evidence indicates that individuals reporting more positive affect are healthier and live longer. The aim of this study was to examine if positive states of mind moderates the effect of perceived stress on psychological functioning and perceived health.

**Methods:**

A cross-sectional sample, n = 382, responded to questions regarding perceived stress, depression, anxiety, perceived health, and frequency of positive states of mind.

**Results:**

Using a series of regression analyses, the results confirmed a moderating role of positive states of mind on the association between perceived stress and psychological outcomes.

**Conclusions:**

Among people experiencing a high frequency of positive states of mind, perceived stress seems to have a low correspondence with depression, anxiety, and perceived health. But among those reporting a low frequency of positive states of mind, perceived stress was more strongly related and depression, anxiety, and perceived health suggesting a buffering effect of positive states of mind against the negative influence of stress.

## Background

There is emerging evidence that people who report higher frequency and intensity of positive affect are healthier and live longer (Xu and Roberts 2010; Wiest et al. 2011). Many studies have demonstrated that measures of subjective well-being are associated with less reported pain (Zautra et al. 2005), better health (Ostir et al. 2001), and mortality (Moskowitz 2003; Moskowitz et al. 2008; Boehm and Kubzansky 2012). Recent studies show that positive affect seems to have a stronger association with health outcome than does negative affect (Ostir et al. 2000; Danner et al. 2001; Ostir et al. 2001; Moskowitz 2003), and more stronger effect than cognitive aspects of subjective well-being (Wiest et al. 2011).

There are several hypothetical pathways through which positive affect might be connected to mental and physical health outcomes. One possible mechanism for the effect of positive affect is through improved self-regulation and improved coping ability, through which positive emotion might function as a buffer against the detrimental effects of stress (Folkman and Moskowitz 2000; Pressman and Cohen 2005; Folkman 2008). An extensive and growing research literature reports on the links between perceived stress and experiences of stressful life events and both negative mental health, such as depression (Hammen 2005), and physical health (Chida et al. 2008). How we handle stressful events and cope with daily stressors could have substantial influence on our well-being and health. According to Lazarus and Folkman’s stress and coping model the activation of coping responses is initiated by an appraisal of an event as harmful, threatening, or challenging (Lazarus and Folkman 1984). In a revision and expansion of this model Folkman emphasizes the importance of positive emotion in the coping process (Folkman 1997; Folkman and Moskowitz 2000), and suggests that positive emotion and positive emotional states can provide a psychological respite from distress that can help sustain continuous coping efforts. There are studies indicating that positive affective states are associated with greater attention to and processing of health-relevant information. Further, according to the broaden-and-build theory, positive affect plays an important role in presenting a wider variety of thought and action alternatives and further enforces people’s general resources (Fredrickson 2004).

Another possible pathway through which positive affect might be connected to health outcomes is through its relationship with health-behaviors and health-behavior change. Behavioral factors such as physical activity, healthy diet (Lyubomirsky et al. 2005), and adherence to medication (Carrico et al. 2010) are possible mediators of the link between positive affect and health outcomes. There are also some evidence that positive affect can facilitate health-behavior change through increased persistence in pursuing health protective goals (Branstrom et al. 2010), and increased and more accurate processing of health-relevant information (Harris and Napper 2005).

In this study we are interested in understanding the association of positive affect measured as frequency of positive states of mind, with psychological functioning and perceived health in an adult, population-based sample in Sweden, and examine the importance of positive states of mind in coping with stress. The buffering effect of frequency of positive states of mind will be examined by analyzing positive states of mind as a moderator of the effects of perceived stress on psychological functioning and perceived health. More specifically, this study was guided by the main research question: Does frequency of positive states of mind moderate the impact of perceived stress symptoms on psychological functioning and perceived health?

## Method

### Study sample and recruitment

In the spring of 2007, a random population based sample of *N* = 1,000 individuals aged 18 – 60 years in Sweden were contacted by mail with a request to participate in the study. The addresses were retrieved from the Swedish Census Registry using random sampling with specifications regarding age range and equal numbers of men and women. Along with the invitation letter, a questionnaire was sent that included self-report measures of perceived stress, overall perceived health, positive states of mind, anxiety, and depression. Those agreeing to participate were encouraged to complete and return the questionnaire in an attached return envelope with pre-paid postage. No compensation for participation was offered, but one mailed reminder was sent to those not responding to the initial invitation. A total of *n* = 382 respondents returned the questionnaire (38% of the target sample). Sample demographics are presented in Table 1. Compared to the total population in Sweden, study respondents were more likely to be women; and have higher education and higher income (all p < 0.001). The study was approved by the Ethics Committee of the Karolinska Institute (No. 2007/48-31/2).

**Table 1:** **Positive states of mind, depression, anxiety, and perceived health by age, gender, education and income**

			Positive states of mind (range: 0 to 4)		Depression (range: 0 to 21)		Anxiety (range: 0 to 21)		Perceived health (range: 0 to 100)	
	n	%	Mean (SD)	Sig.	Mean (SD)	Sig.	Mean (SD)	Sig.	Mean (SD)	Sig.
**Gender**										
Male	140	41.2	2.43 (0.77)	n.s.	3.80 (3.02)	n.s.	6.13 (4.01)	P < 0.01	69.84 (20.25)	n.s.
Female	200	58.8	2.52 (0.81)		3.98 (3.50)		7.35 (4.10)		68.04 (22.26)	
**Age**										
18-29	68	20.1	2.54 (0.76)	n.s.	3.66 (3.35)	n.s.	7.21 (3.71)	P < 0.05 ^a^	68.28 (17.72)	n.s.
30-39	77	22.8	2.40 (0.81)		4.05 (3.43)		7.62 (4.55)		67.64 (21.37)	
40-49	87	25.7	2.40 (0.83)		4.06 (2.13)		7.18 (4.15)		66.48 (23.98)	
50-	106	31.4	2.58 (0.78)		3.77 (3.09)		5.79 (3.83)		71.62 (21.39)	
**Education**										
High school	105	32.0	2.30 (0.78)	P < 0.01 ^b^	4.83 (3.64)	P < 0.001 ^c^	7.22 (4.51)	n.s.	63.46 (24.64)	P < 0.001 ^d^
Some college	122	37.2	2.50 (0.83)		3.70 (3.30)		6.53 (4.15)		70.42 (21.51)	
Bachelors degree or more	101	30.8	2.69 (0.72)		2.85 (2.21)		6.52 (3.32)		74.09 (14.86)	
**Income**										
0 - 29 999 SEK	123	36.9	2.46 (0.75)	n.s.	4.32 (5.50)	P < 0.05 ^e^	7.48 (4.13)	n.s.	65.92 (22.78)	n.s.
30 000–44 999 SEK	104	31.2	2.47 (0.84)		3.81 (3.16)		6.20 (4.02)		70.31 (20.93)	
45 000 SEK or more	106	31.8	2.54 (0.80)		3.22 (2.95)		6.61 (3.93)		71.38 (19.62)	

^a^ Post-hoc analysis showed that differences were only significant between those aged 50 or more as compared to participants aged 30–39.

^b^ Post-hoc analysis showed that differences were significant between those with a Bachelors degree and those with lower education.

^c^ Post-hoc analysis showed that differences were only significant between those with High school and Bachelors degree or more.

^d^ Post-hoc analysis showed that differences were significant between those with High school and those with higher education.

^e^ Post-hoc analysis showed a significant difference between those with an income of 45 000 SEK or more and those with an income of 0–29 999 SEK.

### Psychosocial measures

*Positive States of mind* were measured using the Positive States of Mind (PSOM) scale, a six-item scale measuring positive emotional and cognitive experiences (Horowitz et al. 1988; Adler et al. 1998). It assesses experiences of focused attention, productivity, responsible caretaking, restful repose, sharing, and sensuous nonsexual pleasure during the past week e.g. “Being able to enjoy bodily senses, enjoyable intellectual activity, doing things you ordinarily like, such as listening to music, enjoying the outdoors, lounging in a hot bath”. Responses are indicated on 5-point Likert-type scales from 1 “not at all” to 5 “very much”. Cronbach’s alpha in this study was 0.86. The scale was normally distributed and the mean valued slightly higher than mean values reported for the US (Horowitz et al. 1988).

*Anxiety and Depression* were assessed with the Hospital Anxiety and Depression Scale, a 14 item scale intended for non-psychiatric populations that has been frequently used within healthcare settings (Bjelland et al. 2002). The scale has also been used in community samples and a large population-based study demonstrated that it had adequate psychometric properties (Mykletun et al. 2001). Responses are indicated on 4-point scales from 0 to 3. It consists of two separate subscales measuring current (‘how you feel right now’) state depression (alpha =0.83) and anxiety (alpha = 0.85). The scales were slightly positively skewed but the scales means were comparable with earlier reported data from community samples (Crawford et al. 2001).

*Perceived stress* was assessed with the Perceived Stress Scale (PSS). The PSS is a ten item scale measuring perceptions of stressful experiences during the past month (Cohen et al. 1983). Responses are indicated on 5-point scales from 0 “never” to 4 “very often”. The PSS has previously been used in several different populations. In this sample the internal consistency was 0.86. The scale was normally distributed and had a range from 0 to 40.

*Perceived Health* was measured with two items where the respondents were asked to indicate, on a seven-point scale, their degree of satisfaction with their physical health (‘How would you rate your overall health during the past week?’) and their quality of life (‘How would you rate your overall quality of life during the past week?’) during the past week. The scale is part of the EORTC-QLQ-C30 questionnaire (Aaronson et al. 1993) and constitutes a scale of Global Health with a range from 0 to 100. The scale has been used extensively in health care population but also in large-scale population samples (Michelson et al. 2000). The scale has demonstrated adequate validity in studies comparing patient’s self-assessments with observer’s ratings of open-ended responses to the same questions (Groenvold et al. 1997), and sufficient validity and reliability in psychometric studies of scale structure and internal consistency within scales (Aaronson et al. 1993). In this sample the internal consistency was 0.81, the scale was slightly positively skewed and the mean values were somewhat lower than values reported from a previous population based study in Sweden (Michelson et al. 2000).

### Analysis

Data was analysed using PASW Statistics 18.0. Analysis of variance (ANOVA) procedures were used to test demographic differences in Positive States of Mind, Depression, Anxiety, and Perceived Health scores. Analyses were conducted to examine the potential moderating effect of PSOM on the impact of perceived stress on psychological outcomes such as anxiety and depression, and perceived health. This was done with regression analyses where standardized perceived stress score, standardized PSOM score, and the interaction score for perceived stress and PSOM were entered as independent variables, and depression, anxiety, or perceived health, was entered as a dependent variable. The regression analyses were controlled for age, gender, education and income. To illustrate the moderating effects, figures were constructed with adjusted means of depression, anxiety, and perceived health for groups based on level of perceived stress (tertiles; low, moderate and high), and scores on PSOM (high vs. low based on median). Adjusted means and 95% confidence intervals were calculated using a general linear model (GLM).

To test for common method variance a Harman’s single factor test was conducted and the unrotated factor solution was inspected. This procedure has been suggested as a way to test for common method variance (Podsakoff and Organ 1986), and if a substantial amount of common method variance is present in the data set the result of this test will produce a single factor or one “general” factor accounting for the majority of covariance. In the current data set, the analysis did not give support for a substantial amount of common method variance. The single factor test produced a common factor with an eigenvalue of 12.0 explaining less than half of the variance (37.6%). Further inspection using factor analyses produced five factors, all contributing substantially to the solution (i.e. eigenvalues above 1), corresponding to the five variables entered into the analysis.

## Results

### Descriptive analyses

Demographic differences in frequency of positive states of mind, depression, anxiety and perceived health are presented in Table 1. There were no gender or age differences in frequency of positive states of mind, depression, or perceived health. However, women reported higher degree of anxiety, and respondents 50 years or older reported lower degree of anxiety than younger participants. Higher education was related to higher scores on frequency of positive states of mind, perceived health, and lower scores on depression. Income was negatively related to depression, with significant difference between those with the highest income (45 000 Swedish currency [SEK] or more) as compared to those with the lowest income (0–29 999 SEK).

### The effect of positive states of mind as a moderator of stress

**Figure 1:**
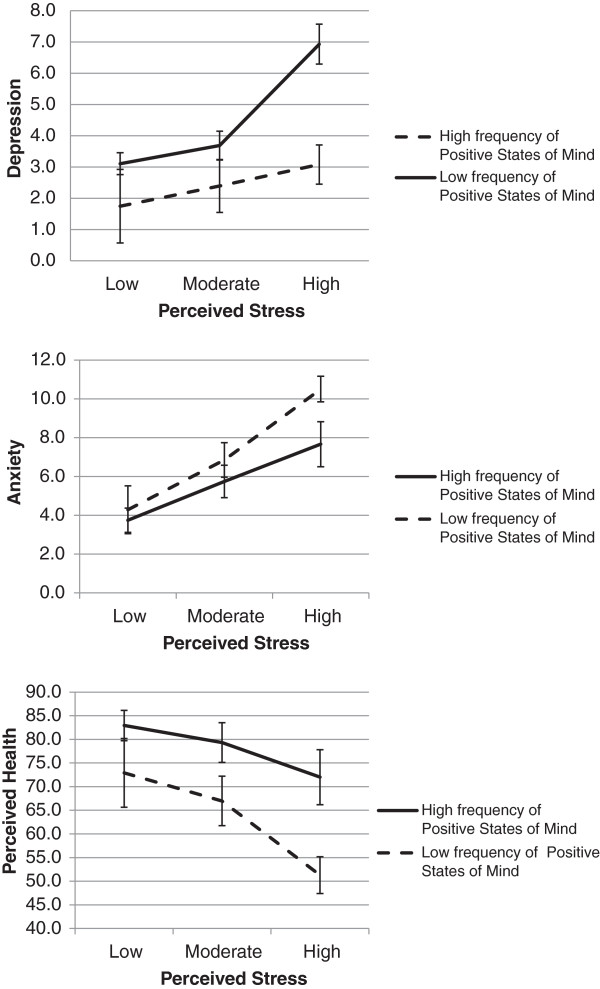
**Mean depression (range 0–21), anxiety (range 0–21), and perceived health score (range 0–100), and 95% confidence intervals are presented to illustrate the moderating effect of Positive States of Mind on the association between perceived stress and depression; anxiety; and global health score.**

Regression analyses showed that perceived stress was strongly associated with the outcome variables and accounted for a substantial portion of variance in depression (β = 0.61, *R*^2^ = 0.34, *F*_(1, 314)_ = 182.60, *p* < 0.001), anxiety (β = 0.71, *R*^2^ = 0.47, *F*_(1, 311)_ = 314.64, *p* < 0.001), and perceived health (β = −0.57, *R*^2^ = 0.30, *F*_(1, 314)_ = 147.17, *p* < 0.001). Positive states of mind was added to the regression analyses and added a significant proportion of explained variance in depression (β = −0.37, *R*^2^ = 0.10, *F*_(1, 313)_ = 60.88, *p* < 0.001), anxiety (β = −0.21, *R*^2^ = 0.03, *F*_(1, 310)_ = 20.70, *p* < 0.001), and perceived health (β = 0.36, *R*^2^ = 0.09, *F*_(1, 313)_ = 51.49, *p* < 0.001). Further analyses testing for the moderating role of PSOM on the association between perceived stress and psychological outcomes showed that the interaction term for the PSOM and perceived stress (PSOM × Perceived stress) accounted for an additional significant proportion of the variance in depression (β = −0.23, *R*^2^_Δ_ = 0.05, *F*_Δ (1, 312)_ = 34.68, *p* < 0.001), anxiety (β = −0.10, *R*^2^_Δ_ = 0.01, *F*_Δ (1, 309)_ = 6.52, *p* < 0.05), and perceived health (β = 0.10, *R*^2^_Δ_ = 0.01, *F*_Δ (1, 312)_ = 5.18, *p* < 0.05). The moderating effects of positive states of mind on depression, anxiety, and perceived health is illustrated in Figure 1.

## Discussion and conclusion

Although the results from this study are based on cross-sectional data, the study gives some support for the importance of positive affect in coping with stress. Among those who reported high frequency of positive states of mind the association between stress and depression, anxiety, and perceived health were diminished. On the other hand, among those with lower frequency of positive states of mind, perceived stress was highly related with increased depression, anxiety, and decreased perceived health. Thus, it seems like higher frequency of positive states of mind make individuals more capable of handling stress without negative consequences for psychological and physical functioning such as depression, anxiety and perceived health. At low levels of stress, this added benefit of positive states of mind is not needed and as a consequence we find no difference in depression, anxiety, or perceived health among these individuals. These results emphasize the importance of considering positive affect in our understanding of the coping process and have implications for the development of stress management interventions.

In this study we use depression, anxiety, and perceived health as outcomes. These measures are conceptually quite distinct but in particular the distinction between anxiety and depression has been discussed considerably (Clark and Watson 1990). In the current study we were interested in examining the differential associations between perceived stress and measures of anxiety, depression, and perceived health. This similarity in findings between these three outcomes is likely the result of both: a) a comparable process in which positive states of mind reduce the impact of stress on all three of these outcomes; b) an overlap between measures of anxiety, depression, and perceived health.

Several previous studies have given support for the importance of positive affect in predicting health outcomes such as morbidity (Ostir et al. 2001) and mortality (Moskowitz 2003; Moskowitz et al. 2008). But the mechanisms behind these associations are not well understood. This study gives some indication of the role of positive states of mind in increasing resilience against stressful events and in strengthening coping ability. There are several possible pathways through which positive emotions and cognitions might influence psychological functioning (Folkman 2008). Positive emotion could increase sustained efforts of cope with stressful situations. Positive states of mind could also give a needed break to restore resourses and alter perceptions of stressful events and situations as more of a challenge than harm or threat.

Future studies of coping with stress should employ longitudinal design and the use of multiple data sources (e.g. diagnostic interviews), and include measures of positive affective states to enable to examine the causal links through which positive emotion and cognitions might lead to increased psychological well-being and perceived health. The study also highlights the possible beneficial effect of including strategies to increase positive affect during stressful conditions, or of using such strategies to prevent poor outcomes following a major stressful event. Such training might strengthen people’s ability to experience high levels of stress without suffering negative psychological and physical health consequences. A recently published pilot study examining the effect of a multiple-component intervention to promote increased positive emotion in individuals experiencing health-related stress, showed promising results in both increasing positive affect and decreasing negative affect (Moskowitz et al. 2011). Future treatment or prevention studies could be designed to experimentally test the influence of strategies and techniques to promote positive affect, and how this relate to changes in well-being and health for people experiencing stress.

While this study contributes to our understanding of individual differences in our reactions to stress, there are several limitations. First, the fact that we used a sample from Sweden reduces our ability to generalize our finding to other countries. Further, we have a substantial selection bias in our recruitment, skewing our sample towards more highly educated women, which further reduces our ability to generalize our findings to the total population. Nonetheless, the study is based on a fairly large community-based sample. This study also suffers from the limitations associated with self-report, including common method variance and socially desirable responding. However, the test for common method variance did not indicate that a substantial amount of common method variance was present in our sample. As with any cross-sectional study, the design of this study limits our ability to make any conclusions regarding causality. The main aim of the current study was to examine the importance of positive affect as a moderator of the association between perceived stress and negative mental and physical outcomes, but the results would have been strengthened if we could have included measures of negative mood in the analyses. We were unable to compare the relative strength of influence of positive vs. negative mood in coping with stress. A further limitation was the self-assessed measure of perceived health, and future studies are needed to understand the impact of positive states of mind on other measures of health e.g. number or severity of physical symptoms.

It is worth noting that the measure of positive experiences used in the current study assesses experiences of Positive States of Mind, this is broader construct than frequency of positive emotions more often used in studies of the influence of positive affect. The limitation of using a measure of Positive States of Mind is that it takes into account a mix of both emotional and cognitive experience, making it difficult to assess the differential influence of the emotional and cognitive content of the positive experiences and its association with other variables. On the other hand, the measure of Positive States of Mind might partly tap into a valuable aspect of positive affect, and is it a very short and easily disseminated measure. Further studies could more in detail examine potential differences in using various measures of positive experiences to evaluate what aspect that is of particular importance for health outcomes.

A key finding of this study is the indication that perceived stress seems to be differentially related to psychological factors at different levels of positive states of mind. Among people experiencing a high frequency of positive states of mind, perceived stress seems to have a low correspondence with depression, anxiety, and perceived health. But among those reporting a low frequency of positive states of mind, perceived stress was more strongly related and depression, anxiety, and perceived health suggesting a buffering effect of positive states of mind against the negative influence of stress. However, to more fully understand the influence of positive emotional experience as a moderator of stress there is a need to replicate this research, and future studies should use a prospective study design and well validated measures of both positive and negative affect and additional measures of health outcome such as number or severity of physical symptoms.
